# Development of a New Ex Vivo Lipolysis-Absorption Model for Nanoemulsions

**DOI:** 10.3390/pharmaceutics11040164

**Published:** 2019-04-04

**Authors:** Lu Xiao, Ying Liu, Tao Yi

**Affiliations:** 1 Department of Basic Medicine, Zunyi Medical University, Zhuhai Campus, Zhuhai 519041, China; xl1527@163.com; 2 Pharmacy Department, Wuhan Medical Treatment Center, Wuhan 430023, China; winter_ling@163.com; 3 School of Health Sciences, Macao Polytechnic Institute, Macau 999078, China

**Keywords:** lipid-based formulations, lipolysis, absorption, poorly water-soluble drugs, model

## Abstract

The use of lipid-based formulations (LBFs) in improving the absorption of poorly water-soluble drugs has now well established. Because the in vivo evaluation of LBFs is labor-intensive, in vitro or ex vivo approaches could provide advantages. In this study, a new ex vivo lipolysis-absorption model (*ev*LAM) composed of an intestinal digestion system and an intestinal tissue system was developed to evaluate and predict the in vivo absorption performances of LBFs. Model factors, including the pH of the system and concentrations of d-glucose and pancreatic lipase, were investigated and optimized by a Box-Behnken design. To evaluate this new model, a lipid formulation of indomethacin, which was chosen based on preliminary studies of pseudo-ternary phase diagrams, emulsion droplets, and solubility, was further investigated by an in vivo pharmacokinetic study of rats, the everted gut sac model, and the *ev*LAM, respectively. The absorption percentages obtained from the *ev*LAM were much more similar to the data of rats in vivo than those from the everted gut sac model, showing a preferable in vitro-in vivo correlation (*r* = 0.9772). Compared with the conventional in vitro and in vivo methods, the *ev*LAM, which allowed precise insights into the in vivo absorption characteristics without much time or a complicated process, could be a better tool for assessing LBFs of poorly water-soluble drugs.

## 1. Introduction

For the oral delivery of poorly water-soluble drugs, lipid-based formulations (LBFs) have gained increasing attention due to enhanced oral bioavailability [1,2]. The main mechanism for the enhanced bioavailability of LBFs [3,4,5,6,7,8] was probably the pre-dissolved state of drugs in LBFs, which could reduce the energy associated with the solid-to-liquid phase transition process and cause the enhanced drug solubilization by colloidal structures. The formations of colloidal structures were the results of interactions among LBFs, their digestion products, and endogenous surfactants such as bile salts and phospholipids [9,10,11,12,13]. The powerful digestive system in the intestine could play an important role in the fate of LBFs [14,15].

It is very important to provide a fast and accurate method to evaluate the in vitro and in vivo characteristics of LBFs. Because the in vivo pharmacokinetics study is expensive and labor-intensive, the evaluation of LBFs by in vitro or ex vivo assays could present important advantages. Conventional in vitro methods for screening formulation and evaluating characteristics of LBFs are based on the pseudo-ternary phase diagram, the comparison of droplet size and solubility, and the in vitro lipolysis [16,17]. Phase diagram, emulsion droplet size, and in vitro solubility assays are important for the preliminary choice of a lipid-based formulation, especially for microemulsions and self-microemulsifying drug delivery systems. However, the in vitro findings in these assays only correlate poorly with the in vivo absorption characteristics. 

The in vitro lipolysis model for assessing the fate of drugs of LBFs, whether they were soluble or precipitated in the intestinal digestive system, has been well-recognized [18,19,20,21,22,23,24]. The standard in vitro lipolysis assay was performed using a pH-stat to maintain the pH of the system, adding porcine pancreatin to serve as a lipase-colipase model for human pancreatic juice, and using bile salt-lecithin mixed micelles to provide a solubilization environment. The data generated from the pH-stat could be used to quantify the rate and extent of lipolysis through recording the amounts of free fatty acids released from LBFs. After the reaction had been terminated, the products of lipolysis could be examined to determine the fate of the drug after lipolysis [5,19]. The in vitro lipolysis model is useful for the optimization of LBFs and has been used for lipid nanoparticles [25]. Since the pancreatic extract contained both the pancreatic lipase and carboxyl ester hydrolase, the in vitro lipolysis model was improved by using porcine pancreatic extract, as it was therefore permitted to mimic the duodenal digestive lipolysis in a biorelevant manner. However, the in vitro lipolysis model may not be predictive for actual in vivo absorptions due to the lack of effective simulation of the internal physiological environment. However, the in vitro lipolysis model may not be predictive for actual in vivo absorptions due to the lack of effective simulation of the internal physiological environment [24]. 

The everted gut sac model (EGSM), commonly using intact intestinal mucosal epithelium of rats to mimic the in vivo conditions, has been widely used to pharmacokinetic studies such as drug absorption, drug metabolism in intestinal segments, efflux transport, multidrug resistance, and drug interactions [26]. The viability of intestinal segments under in vitro conditions was impacted by experimental factors such as pH, aeration, temperature, and the concentration of the substance. The EGSM provides a relatively large surface area available for absorption and a mucus layer. Consequently, results from EGSM have been in agreement with in vivo findings for many drugs [26]. However, the EGSM could not accurately evaluate LBFs due to the lack of the simulation of the lipolytic condition. Therefore, it was hypothesized that the combination of the EGSM and the in vitro lipolysis model could form a new ex vivo model which should be much closer to the in vivo conditions and, resultantly, could evaluate LBFs more accurately. 

The first aim of this study was to establish a new ex vivo lipolysis-absorption model (*ev*LAM) for evaluating and predicting the in vivo performance of LBFs. In this study, the *ev*LAM composed of an intestinal digestion system (a pH-stat to maintain the pH of the system, adding porcine pancreatin to serve as a lipase-colipase model for human pancreatic juice, and using bile salt-lecithin mixed micelles to provide a solubilization environment) and an intestinal tissue system (intestinal segments under physiological medium to obtain absorption data). The new *ev*LAM was much closer to the in vivo conditions and resultantly could evaluate LBFs more accurately. Model factors, including the pH of the system and concentrations of Ca^2+^, d-glucose, K^+^, and pancreatic lipase, were investigated and optimized by a three-level Box-Behnken design. The pH of the system, concentrations of d-glucose, and pancreatic lipase were chosen as the independent variables; the intestinal tissue activity and the fatty acid concentration were the dependent variables [19,27,28]. 

Furthermore, the in vitro absorptions obtained from the new *ev*LAM and the conventional EGSM were compared with the pharmacokinetics data of rats. The in vitro-in vivo correlations of absorption curves obtained from the two models were further compared to indicate the advantages of the new model in evaluating and predicting the in vivo performance of LBFs.

## 2. Experimental Section 

### 2.1. Materials

Sodium taurodeoxycholate (97%), porcine pancreatin (8 × USP specifications activity), and Trizma maleate (99.5%) were purchased from Sigma Chemical Co. (St. Louis, MO, USA). Medium chain mono- and di-glyceride (Capmul MCM) were kindly donated by Abitec Co. (Janesville, WI, USA). Lecithin (approximately 80% pure phosphatidylcholine) was a gift from Q.P. Co. (Fuchu-Shi, Tokyo, Japan). Indomethacin (99.5%) was purchased from Zizhu Pharmaceutical Co. (Beijing, China). The Naproxen sodium reference substance (99.9%) and indomethacin reference substance (99.9%) were purchased from the China Institute for the Control of Drugs and Biological Products (Beijing, China). A lactate dehydrogenase Assay Kit was purchased from the Nanjing Jiancheng Bioengineering Institute (Nanjing, China). Other chemicals were of HPLC or analytical grade. 

### 2.2. Preparation of the Intestinal Tissue Medium in the Fasted State 

1.25 mM of lecithin was dissolved in chloroform in a round bottom flask, and then chloroform was evaporated off under vacuum, resulting in a thin film of lecithin around the bottom of the flask. After the addition of 5 mM of sodium taurodeoxycholate, 50 mM of Trizma maleate, and 150 mM of NaCl and Ca^2+^ solution (at 1, 3, 5 or 10 mM), the mixed solution was adjusted with NaOH or HCl to a pH of 7.500 ± 0.001 and then stirred and equilibrated for 12 h; after which, it finally formed a clear and slightly yellow solution. At last, d-glucose solution (at 0, 5, 10 or 15 mM) and K^+^ solution (at 0, 3.5, 5.5 or 6.5 mM) were added in before use.

### 2.3. Preparation of Intestinal Segments

All surgical and experimental procedures were approved by the Animal Research Ethics Committee of Zunyi Medical University (No.: ZMCER2018A051). Male Wistar rats (Chongqing, China) 11–12 weeks old, 250 ± 20 g in weight, and fasted for 24 h prior to the experiment were anaesthetized intraperitoneally with 3.5% chloral hydrate (1 mL·100 g^–1^). The small intestine was removed out and washed three times with saline (0.9% NaCl solution) at 37 °C. The intestine was immediately placed in an oxygenated (O_2_: CO_2_ = 95:5% *v*/*v*) intestinal tissue medium (pH 7.5, 37 °C). The intestinal segment (5–7 cm in length) was everted on a tube (2.5 cm in diameter), and then one end was sealed with a clamp.

### 2.4. Establishment of the evLAM

**Figure 1:**
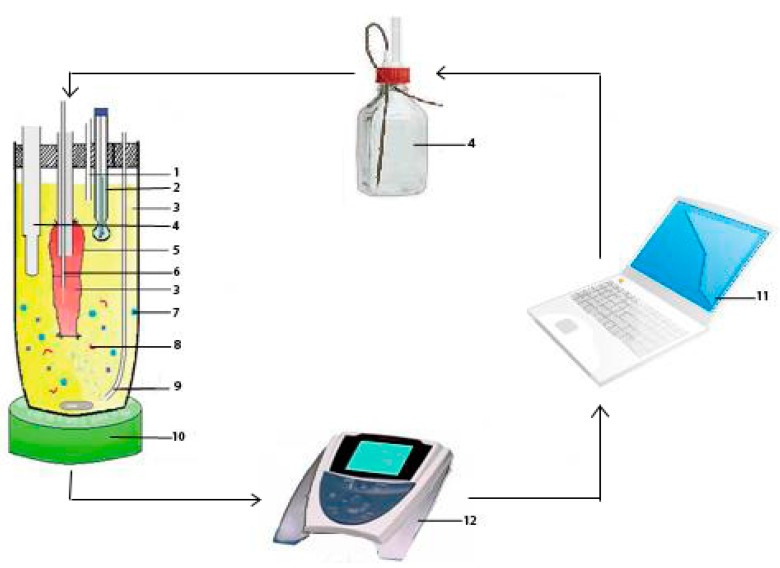
The new lipolysis-absorption model for lipid-based formulations: (**1**) Vent; (**2**) pH electrode; (**3**) intestinal tissue medium; (**4**) NaOH autoburette; (**5**) intestinal segment; (**6**) sampler; (**7**) a lipid-based formulation; (**8**) pancreatic lipase/colipase; (**9**) O_2_/CO_2_; (**10**) temperature controlled stirrer; (**11**) computer; and (**12**) pH-stat meter controller.

A new *ev*LAM, composed of an intestinal tissue system (intestinal segment, medium, temperature controlled stirrer, vents, and O_2_/CO_2_) and an intestinal digestion system (pH-stat meter controller, NaOH autoburette, pH electrodes, and computer) was set up based on characteristics of intestinal digestion of LBFs. As shown in Figure 1, the intestinal segment was filled with fresh and oxygenated intestinal tissue medium using a 1 mL glass syringe and then incubated in a centrifuge tube containing oxygenated intestinal tissue medium with the lipid-based formulation to be assayed at 37 °C. Lipase and pancreatin were used to mimic the intestinal digestive lipolysis in a biorelevant manner. Then, the pancreatic lipase extract, which was prepared by adding 1 g of porcine pancreatic lipase powder into 5 mL of digestion buffer (Trizma maleate, NaCl, Ca^2+^, pH 7.5) and stirred for 15 min followed by centrifugation at 1,600× g and 5 °C for 15 min, was added to initiate lipolysis. In the process of the lipolysis, the pH of the system was sustained by a pH-stat automatic titration unit with 0.2 M NaOH. At designated intervals of 2 h experiment, samples of 200 μL were collected from the gut sac and conserved at −20 °C until analysis. Each experiment was performed by three parallel treatments, and the average value was used. At the same time, the fresh intestinal tissue medium with the same volume was added. 

### 2.5. Optimization of the evLAM

#### 2.5.1. Measurement of the Attenuation Rate of Intestinal Tissue Activity

The concentrations of lactate dehydrogenase in the sample at every time point was measured by the lactate dehydrogenase assay kit. The determination was performed three times, and the average value was used. The attenuation rate (AR) of intestinal tissue activity was calculated as follows.

(1) $$\mathrm{AR}=({\mathrm{CLDH}}_{\mathrm{end}}-{\mathrm{CLDH}}_{0})/\mathrm{time}\;\mathrm{interval}$$

where CLDH_end_ was the concentration of lactate dehydrogenase at the end, and CLDH_0_ was the concentration of lactate dehydrogenase at the beginning. The release of lactate dehydrogenase increased with the increasing damage of intestinal tissue. The faster intestinal tissue activity decreased, the faster the attenuation rate was.

#### 2.5.2. Analysis of the Amount of Fatty Acids

The release of free fatty acids from the lipolysis of LBFs was monitored using a titration method [29]. The amount of fatty acids in each sample was determined by the end-point titration with 0.2 M of NaOH. The determination was performed by three parallel treatments, and the average value was used.

#### 2.5.3. Three-Level Box-Behnken Design

A three-level Box-Behnken design comprised of 15 experimental runs was constructed by Design-Expert (Version 8.0.0, Stat-Ease Inc., Minneapolis, MN, USA) [30]. Independent variables and dependent variables are listed in Table 1 along with their low, medium, and high levels and target value, which were selected based on results from preliminary experiment.

### 2.6. Evaluation of the evLAM

#### 2.6.1. Choosing a Lipid-Based Formulation of Indomethacin

A lipid formulation of indomethacin was chosen as follows: Solubility and pseudo-ternary phase diagrams were first studied to obtain four formulations, Formulation I to IV (shown in Supplementary Figures S1 and S2). Then, in vitro characteristics of these four formulations, such as the droplet size, self-emulsifying efficiency, and solubility, were determined (shown in Supplementary Table S1). Based on the results, the optimal formulation of indomethacin, Formulation II composed of Labrafac@Lipohile WL1349, Cremophor RH40, and Transcutol P (20:60:20, *w*/*w*), was chosen for further studies. The solubility of indomethacin in Formulation II was 32.19 mg/g, and the drug content of indomethacin in Formulation II was 16.0 mg/g. After lipolysis, the proportion of drug dispersed in aqueous phase and precipitation phase was 86.95 ± 0.75% and 12.50 ± 0.26%, respectively.

#### 2.6.2. HPLC Analysis of Indomethacin

The concentration of indomethacin was determined by an HPLC analysis system (Agilent 1200, Agilent Technologies, Santa Clara, CA, USA) with an Agilent ODS-C18 column (250 × 4.6 mm, 5 μm). The column temperature was 25 °C, and the injection volume was 10 μL. The mobile phase was a mixture of acetonitrile and 0.1 M sodium acetate at a ratio of 40:60 (*v*/*v*), with pH of 5.0 adjusted by acetic acid. The detection was carried out at a wavelength of 320 nm, with a flow rate of 1.0 mL/min. The percent relative standard deviation (RSD%) of method precision was lower than 2%. The observed-to-expected ratios for spiking recovery ranged from 100.8% to 101.1%, showing the acceptable accuracy of the method. The sensitivity of the method represented by limit of quantitation was 0.1 μg/mL.

#### 2.6.3. Comparison of the In Vitro Absorption between evLAM and EGSM

The *ev*LAM optimized above was used to investigate the in vitro intestinal absorption of indomethacin of Formulation II. Briefly, a known quantity of Formulation II (250 mg formulation per 10 mL intestinal tissue medium) was crudely emulsified in the intestinal tissue medium in a centrifuge tube. Lipolysis was initiated by the addition of pancreatic lipase extract into the gut sac. Samples were collected at designated intervals, and drug concentrations were measured by HPLC as described above. The in vitro cumulative absorption percentage ($P_{a}$) was calculated as follows:

(2) $$P_{a}=\frac{(V_{\mathrm{mea}}\times C_{n}\times \frac{V_{\mathrm{bal}}}{V_{\mathrm{sam}}}+V_{\mathrm{mea}}\times \sum_{t=1}^{n-1}C_{i})/V}{C}\times 100\%$$

where *C*_n_ was the drug concentration of each sample; *V*_bal_ was the volume of the intestinal tissue medium before balance; *V*_sam_ was the volume of samples collected at each time point; *V*_mea_ was the volume of samples measured at each time point; *V* was the total volume of the intestinal tissue medium in the gut sac; and *C* was the initial drug concentration. 

The conventional EGSM was also used to study the in vitro intestinal absorption of indomethacin of Formulation II. After anesthesia of rats, an intestinal segment (about 6 cm) was removed rapidly. The segment was washed with saline at 4 °C and everted over a glass rod gently. One end of the everted segment was tied with suture, and then the intestinal segment was filled with Krebs solution at 37 °C by needle tubing. The other end of the filled intestinal segment was hanged up with a tie. Finally, the intestinal segment was put into a beaker containing 20 mL medium with Formulation II at 37 °C. Samples of 0.1 mL were collected at designated intervals, and the drug concentrations were measured by HPLC as described above. The blank medium of 0.1 mL was replenished at each time point. The value of $P_{a}$ was also calculated as described above. 

#### 2.6.4. In Vivo Absorption Study of Indomethacin LBF

A pharmacokinetic study was designed to investigate the in vivo absorption of the indomethacin LBF, Formulation II. Five Male Wistar rats (250 ± 10 g), which had been acclimatized for at least 1–2 weeks before the experiment, were fasted for 24 h prior to drug administration but allowed free access to water. Formulation II was administered intragastrically to each rat at a dose of 4.5 mg·kg^–1^ of indomethacin. About 200 μL of blood sample was collected from the rat tail vein into heparinized tubes at designated time intervals [31]. Plasma was separated by centrifugation and stored at −20 °C until analysis.

The concentration of indomethacin in plasma was determined by HPLC [32] as follows: 10 μL of internal standard solution (200 mg·L^–1^ naproxen sodium solution) was added into 150 μL of plasma and mixed for 5 min. Then, 15 μL of a phosphate buffer (pH 7.0) and 20 mg of NaCl were added. The sample was extracted with 375 μL acetidin by vortex-mixing for 10 min and centrifuging at 10,000× *g*. The supernatant was transferred to a clean tube and evaporated by nitrogen purging. The residue was reconstituted in 50 μL methanol. After vortex-mixing for 10 min, 20 μL of the sample was used for HPLC as described above. The 3p97 computer program and a Wagner-Nelson method were employed to analyze the plasma concentration-time data. The in vivo absorption percentage (*f*_a_) was calculate as follows.

(3) $$f_{a}=\frac{C_{t}+K_{e}\int_{0}^{\tau }C_{t}dt}{K_{e}\int_{0}^{\infty }C_{t}dt}\times 100\%$$

where *C*_n_ was the drug concentration at each time point, and *K*_e_ was the elimination rate constant.

### 2.7. Statistical Analysis 

All data were expressed as mean ± SD. Statistical analysis and data fitting were performed using SPSS 16.0 (SPSS Inc., Chicago, IL, USA). One-way analysis of variance (ANOVA) was performed to test differences for statistical significance. Difference between mean values was considered statistically significant at *p* < 0.05 and very statistically significant at *p* < 0.001.

## 3. Results and Discussion

### 3.1. Effects of Components of the evLAM on Activity of Intestinal Tissue

As a new model for assessing the in vitro absorption of LBFs, it was important to simulate the actual dynamics of intestinal fluids. Because the viability of intestinal tissue under in vitro conditions was impacted by many factors, it was also important to select components of this new *ev*LAM based on the minimal tissue damage. Components of the *ev*LAM and their concentration ranges were all chosen as a compromise between in vivo values as the literatures reported [27,28,33,34,35,36,37] and pre-experiments in our laboratory. Trizma maleate was chosen at the concentration of 50 mM, which was similar to that used in other studies [19,38,39,40,41,42]. Sodium taurodeoxycholate and phosphatidylcholine were both added because that the administered exogenous lipids and their digestion products could intercalate into endogenous sodium taurodeoxycholate and phosphatidylcholine structures, changing the nature of solubilizing species, promoting micelle swelling and further increasing solubilization capacity [10,15,33]. Concentrations of sodium taurodeoxycholate and phosphatidylcholine in intestinal fluid after oral administration of LBFs have not been reported, so 5 mM of sodium taurodeoxycholate and 1.25 mM phosphatidylcholine were chosen in the *ev*LAM based on their typical concentrations in the fasted state as the literatures reported [10,15,33].

**Figure 2:**
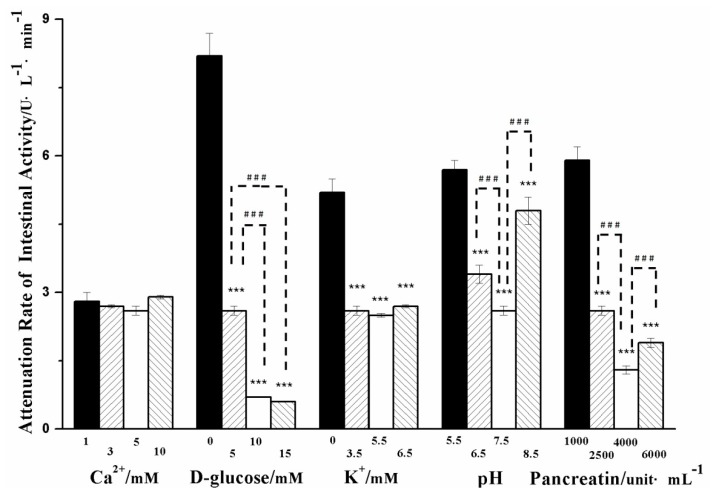
Influences of Ca^2+^, d-glucose, K^+^, pH, and pancreatic lipase on the attenuation rate of the intestinal tissue activity of the ex vivo lipolysis-absorption model. (Mean ± S.D., *n* = 5). *** very statistically significant (*p* < 0.001) compared with D-glucose of 0 mM, K^+^ of 0 mM, pancreatic lipase of 1000 uint·mL^–1^ and pH of 5.5, respectively. ^###^ very statistically significant (*p* < 0.001) difference in pairwise comparison.

Influences of Ca^2+^, d-glucose, K^+^, pH, and pancreatic lipase on the activity of intestinal segments in the *ev*LAM were investigated. Value ranges of these model factors were set up based on the literatures. The same amount of Formulation II was used in the *ev*LAM with different levels of model factors. Attenuation rates of the activity of intestinal segments under different conditions were measured and are shown in Figure 2. 

Though there was no mention of a calcium binding site in the 3-D structure of pancreatic lipase/co-lipase complex [34,35], Ca^2+^ was necessary for the activity of pancreatic lipase. The formation of Ca^2+^-soaps could draw the equilibrium towards the ionized fatty acids and maintain lipolysis in the presence of bile [36]. The mean concentration of Ca^2+^ in the fasted state was 0.5–3 mM in the duodenum, and Ca^2+^ of 1–10 mM was investigated in the previous studies [27,28,37]. As shown in Figure 2, no significant differences in the attenuation rate of intestinal tissue activity were seen in a range of Ca^2+^ concentrations, which suggested that Ca^2+^ did not directly influence the activity of intestinal tissue. 

The attenuation rate of intestinal tissue activity decreased with the increasing concentration of d-glucose in the range of 0–15 mM, suggesting that the higher concentration of d-glucose could maintain the activity of intestinal tissue for a much longer time. It was probably due to that d-glucose was the energy source for cell metabolism. 

The attenuation rate of intestinal tissue activity was significantly decreased after adding K^+^. This could be due to the fact that the Na^+^ pump was usually activated upon K^+^, benefitting the secondary active transport of d-glucose [43] and providing more energy to intestinal tissue cells. However, when the amount of K^+^ was sufficient in the concentration range of 3.5–6.5 mM, there were no significant changes in attenuation rate of intestinal tissue activity. 

The pH value of the in vitro lipolysis model showed a high variability ranging from 5.8 to 8.5 [27,28]. As shown in Figure 2, the decreased attenuation rate of intestinal tissue activity appeared significantly at the pH range of 5.5 to 7.5, but the increased attenuation rate of intestinal activity was observed at pH 8.5. It was suggested that it was important for the activity of intestinal tissue to select a moderate pH.

The pancreatic lipase had an activity of 500–600 unit·mL^–1^ in the fasted state and 800–1800 unit·mL^–1^ in the fed state, respectively [14,34,35]. In some previous studies, the pancreatic lipase reached up to 10,000 unit·mL^–1^ [19]. As shown in Figure 2, the attenuation rate of intestinal tissue activity was significantly decreased when the pancreatic lipase concentration increased from 1000 to 4000 unit·mL^–1^, but the attenuation rate increased when the pancreatic lipase reached up to 6000 unit·mL^–1^. It was suggested that excessive pancreatic lipase might damage intestinal tissue. 

From the results above, it could be seen that the pH of the system, concentrations of d-glucose, and pancreatic lipase had much more influence on the activity of intestinal tissue than the other model factors. Therefore, these three factors were chosen as independent variables of Box-Behnken design for optimizing the *ev*LAM.

### 3.2. Optimization of the evLAM by Box-Behnken Design

All the responses observed for 15 experimental runs were simultaneously fitted to first order, second order, and quadratic models by Design Expert 8.0. It was observed that the best-fitted model was the quadratic model. Comparative values are given in Table 2 along with the regression equations generated for each response. Only statistically significant (*p* < 0.05) coefficients were included in the equations.

A positive value indicated an effect that favored the optimization, while a negative value represented an inverse relationship between the factor and the response. As shown in Table 2, it was evident that the pH of the system (*X*_1_) had positive effects on the two responses—the attenuation rate of intestinal tissue activity (*Y*_1_) and the amount of fatty acids (*Y*_2_). Concentrations of d-glucose (*X*_2_) and pancreatic lipase (*X*_3_) had positive effects on the amount of fatty acids but had negative effects on the attenuation rate of intestinal tissue activity. More than one factor term or the coefficients with higher order terms in the regression equation represented, respectively, interaction terms or quadratic relationships, which suggests that the relationships between factors and responses were not always linear. As shown in Table 2, the interaction effect of concentrations of d-glucose and pancreatic lipase was only positive for the attenuation rate of intestinal tissue activity, but interaction effects between pH and d-glucose concentration, pH, and pancreatic lipase concentration were unfavorable for the amount of fatty acids. Higher and positive quadratic effects of pH and d-glucose concentration were observed for both the attenuation rate of intestinal tissue activity and the amount of fatty acids. Quadratic effects of pancreatic lipase concentration were positive or negative for the attenuation rate of intestinal tissue activity and the amount of fatty acids, respectively. 

**Figure 3:**
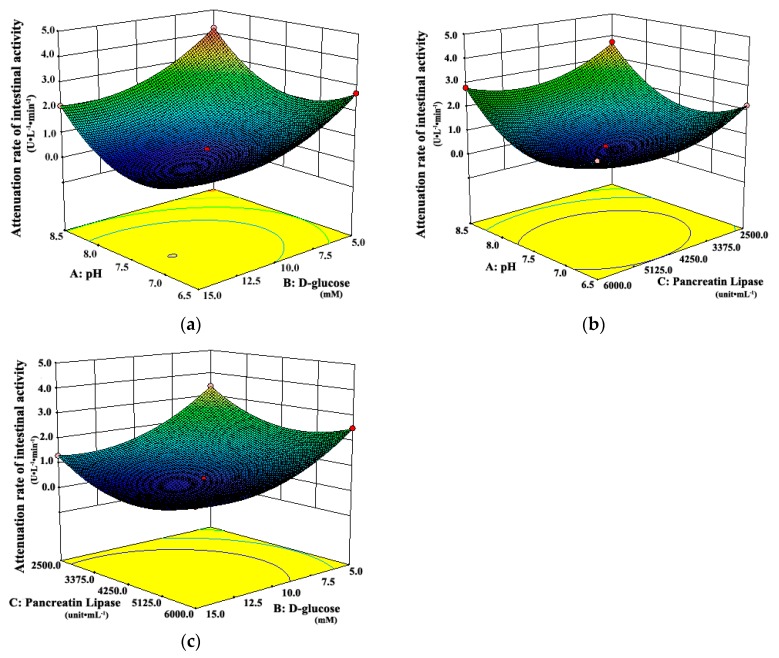
Response surface plots showing interaction effects of pH, d-glucose, and pancreatic lipase on the attenuation rate of intestinal tissue activity when (**a**) pancreatic lipase, (**b**) d-glucose, and (**c**) pH held constant, respectively.

**Figure 4:**
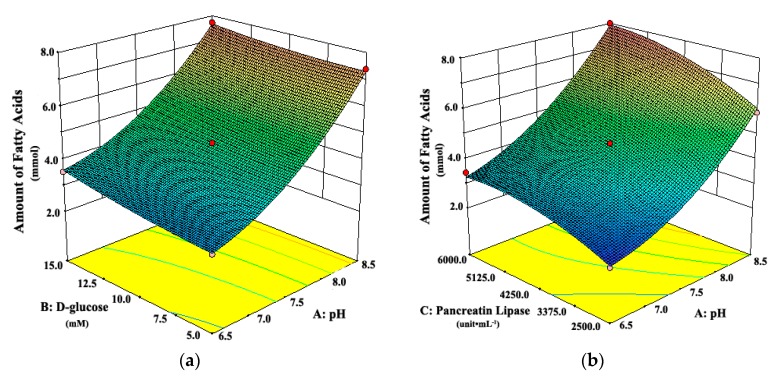
Response surface plots showing interaction effects of pH, d-glucose, and pancreatic lipase on the amount of fatty acids from digestion when (**a**) pancreatic lipase or (**b**) d-glucose held constant, respectively.

Three-dimensional response surface plots, which could represent interactions of all factors on the responses more clearly, are shown in Figure 3 and Figure 4. When the third factor was kept at a constant level, these plots were very useful in study of the effects of another two factors on the response at the same time.

As shown in Figure 3a, when the pH of the system was 6.5 or 8.5, the attenuation rates of intestinal tissue activity were higher than those at other moderate levels of pH, regardless of the d-glucose concentration. A similar observation can be seen in Figure 3b. The attenuation rate of intestinal tissue activity increased when the pH of the system changed from the middle to the both ends of the range of 6.5 to 8.5, whether at low or high concentration of pancreatic lipase. It suggested that the moderate pH was favorable for the intestinal tissue activity, which was due to that the moderate pH simulated the physiological environment of intestinal tract in vivo. However, Figure 4 shows that the amount of fatty acids increased always with the increasing pH, which suggests that the high pH could promote the in vitro lipolysis. It might be due to that pancreatic lipase showed the highest catalytic activity in vitro around pH of 8 [35,40,41,42]. In the literature [28,35,40,41,42], the pH was usually set about 7.4, which was the optimum value for the intestinal cell culture and the best activity of pancreas lipase. 

Figure 3a shows that the attenuation rate of intestinal tissue activity decreased as d-glucose concentration increased from 5.0 mM to 15.0 mM, whether at low or high level of pH. A similar observation can be seen in Figure 3c. The attenuation rate of intestinal tissue activity decreased with the increasing concentration of d-glucose, whether at low or high concentration of pancreatic lipase. The possible explanation was that more energy source from d-glucose was provided for intestinal tissue cells at higher concentrations of d-glucose, which could maintain the activity of intestinal tissues well in vitro. Moreover, Figure 4a shows that there was a slight increase in the amount of fatty acids with the increase of d-glucose concentration, whether at low or high level of pH. All the results above suggested that the high concentration of d-glucose was favorable for the *ev*LAM.

As shown in Figure 3b, the attenuation rate of intestinal tissue activity would be at the least level when the concentration of pancreatic lipase was in the middle of the range of 2500 to 6000 unit·mL^–1^, whatever the pH of the system was. Figure 3c shows the similar variation trends in the attenuation rate of intestinal tissue activity with the concentration of pancreatic lipase, whether at low or high concentration of d-glucose. However, Figure 4b shows that the amount of fatty acids increased with the increasing concentration of pancreatic lipase, which suggested that the high concentration of pancreatic lipase could promote the in vitro lipolysis. Thus, the optimal concentration of pancreatic lipase should be a compromise between the optimal conditions in vitro for the intestinal tissue activity and the lipolysis.

The optimum *ev*LAM was selected based on the criteria for attaining the optimum value of the model by applying constraints on the attenuation rate of intestinal tissue activity (minimum) and the amount of fatty acids (4.468 ≤ *Y*_2_ ≤ 4.703). Based on ‘trading of’ various response variables and comprehensive evaluation of feasibility search and exhaustive grid search, the *ev*LAM with pH of 7.37, d-glucose of 12.06 mM, and pancreatic lipase of 4.94 × 10^3^ unit·mL^−1^ was found to fulfill the optimum model. The predicted values and the observed values were in reasonably good agreement (Table 3).

### 3.3. Evaluate of the evLAM by the Pharmacokinetics in Rats

**Figure 5:**
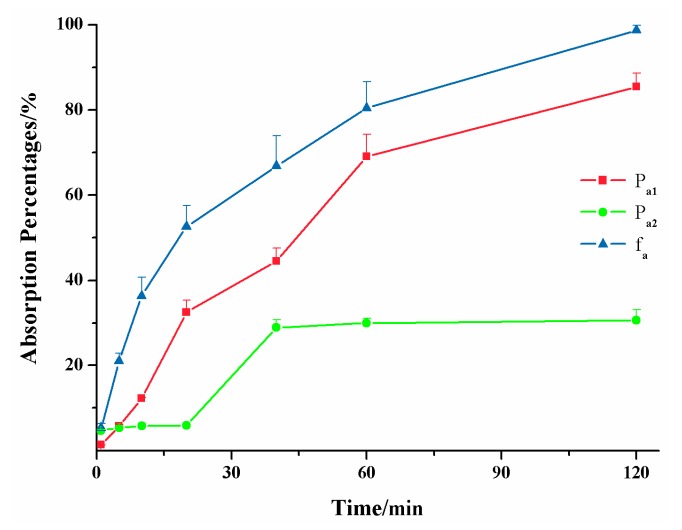
Absorption percentages of the lipid-based formulation of indomethacin from the new ex vivo lipolysis-absorption model (■, *P*_a1_), the conventional everted gut sac model (●, *P*_a2_) and pharmacokinetics test of rats (▲, *f*_a_) within 2 h. (*n* = 5).

The first aim of this study was to establish the ex vivo lipolysis-absorption model for evaluating and predicting the in vivo performance of LBFs. We conducted the in vivo pharmacokinetic studies to obtain the real absorption of formulations in vivo. Figure 5 shows that absorption percentages obtained from the new *ev*LAM (*P*_a1_) were far higher than those from the conventional EGSM (*P*_a2_) within 2 h. The EGSM provided a relatively large surface area available for absorption and a mucus layer [26] but lacked the simulation of lipolytic condition, resulting in the inadequate absorption in vitro of LBFs. On the contrary, the *ev*LAM provided the almost real environment for the digestion of LBFs. Therefore, the in vitro absorption of poorly water-soluble drugs in LBFs could be obtained more accurately by the *ev*LAM.

**Figure 6:**
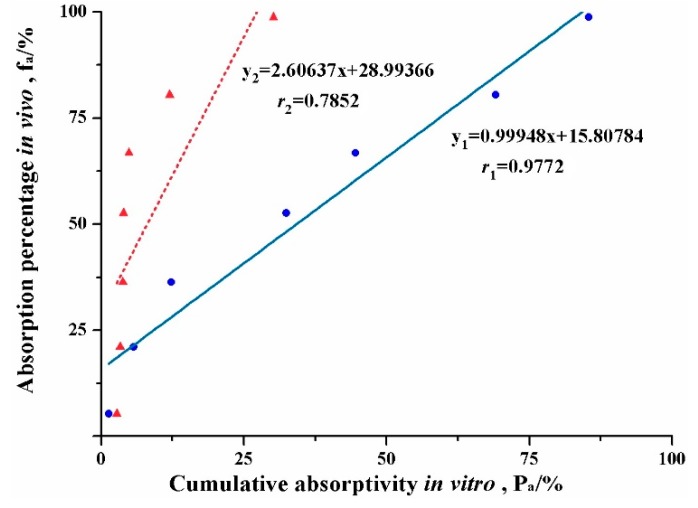
Plots of the in vivo-in vitro correlation of absorption curve for the lipid-based formulation of indomethacin obtained from the conventional everted gut sac model (▲) and the new ex vivo lipolysis-absorption model (●), respectively.

Figure 6 shows the in vitro-in vivo correlations (IVIVC) of absorption curve obtained from the new *ev*LAM and the conventional EGSM. The regression correlation coefficient (*r*) of IVIVC for the *ev*LAM (*r*_1_ = 0.9773, *n* = 7) was far higher than the critical correlation coefficient (*r* = 0.8740, *P* < 0.01, *n* = 7), while r for the EGSM (*r*_2_ = 0.7852, *n* = 7) was below the critical correlation coefficient. Therefore, there was a significant correlation between the absorption curve from the *ev*LAM and the in vivo absorption curve of rats. It was suggested that the *ev*LAM possessed good ability to predict the in vivo performance of lipid formulations. On the contrary, there was no significant correlation between the absorption curve from the EGSM and the in vivo absorption curve of rats. Indeed, these results demonstrated that the *ev*LAM, allowed precise insights into the in vivo absorption characteristics of LBFs, which suggests that it should be an attractive and great potential method for screening formulation and evaluating characteristics of LBFs.

## 4. Conclusions

In this paper, a new *ev*LAM was developed to predict the intestinal absorptions of poorly water-soluble drugs in LBFs. This new model was composed of an intestinal digestion system and an intestinal tissue system. d-glucose, pancreatic lipase, and pH significantly affected the in vitro activity of intestinal tissue and the in vitro lipolysis. The optimal model parameters by the Box-Behnken design were set up as follows: a pH of 7.37, d-glucose of 12.06 mM, and a pancreatic lipase of 4.94 × 10^3^ unit·mL^−1^. For a typical lipid-based formulation, absorption percentages obtained from the optimal *ev*LAM showed a much better IVIVC with absorption percentages of rats in vivo. The new *ev*LAM could make up for the inadequacy of conventional methods and be a better tool for assessing LBFs of poorly water-soluble drugs. 

## Figures and Tables

**Table 1:** Variables and their levels in the Box-Behnken design.

Factor	Levels used, Actual (Coded)		
	Low (–1)	Medium (0)	High (+1)
Independent variables			
*X*_1_ = the pH of the system	6.5	7.5	8.5
*X*_2_ = d-glucose concentration (mM)	5	10	15
*X*_3_ = pancreatic lipase concentration (unit·mL^–1^)	2500	4250	6000
Dependent variables	Target value		
*Y*_1_ = Attenuation rate of intestinal tissue activity (U·L^–1^·min^–1^)	Minimize		
*Y*_2_ = Amount of Fatty Acids (mmol)	4.468~4.703		

**Table 2:** Regression analysis for responses for fitting to quadratic model.

	*R* ^2^	Adjusted *R*^2^	Predicted *R*^2^	SD	% CV
*Y*_1_	0.9999	0.9996	0.9982	0.026	1.31
*Y*_2_	0.9966	0.9921	0.9656	0.16	3.32
Regression equations of the fitted quadratic model					
*Y*_1_ = 0.40 + 0.69*X*_1_ − 0.99*X*_2_ − 0.40*X*_3_ − 0.08*X*_1_*X*_2_ − 0.049*X*_1_*X*_3_ + 0.064*X*_2_*X*_3_ + 1.33*X*_1_^2^ + 0.74*X*_2_^2^ + 0.82*X*_3_^2^					
*Y*_2_ = 4.55 + 2.13*X*_1_ + 0.27*X*_2_ + 0.75*X*_3_ − 0.089*X*_1_*X*_2_ − 0.20*X*_1_*X*_3_ + 0.72*X*_1_^2^ + 0.10*X*_2_^2^ − 0.41*X*_3_^2^					

**Table 3:** Optimized values obtained by the constraints applied on the attenuation rate of intestinal tissue activity (*Y*_1_) and the amount of fatty acids (*Y*_2_) (Mean ± S.D., *n* = 5).

Variable	Nominal Values	Response	Predicted Values	Observed Values
*X*_1_ (the pH of the system)	7.37	*Y*_1_ (U·L^–1^·min^–1^)	0.041	0.047 ± 0.002
*X*_2_ (d-glucose concentration)	12.06 (mM)	*Y*_2_ (mmol)	4.629	4.355 ± 0.720
*X*_3_ (pancreatic lipase concentration)	4.94 × 10^3^ (unit·mL^−1^)			

## Supplementary Materials

The following are available online at https://www.mdpi.com/1999-4923/11/4/164/s1, Figure S1. Pseudo-ternary Phase Diagrams: “Me” represented microemulsion area; (A) the mixing ratio of Cremophor RH40 and Transcutol P was 3:1; (B) the mixing ratio of Cremophor RH40 and Transcutol P was 1:1; (C) the mixing ratio of Cremophor RH40 and Transcutol P was 1:3. Figure S2. The microscopic images of the four formulations. Formulations were showed as labeled and the ruler was in the bottom right corner of the diagram. Table S1. Self-emulsifying Time, droplet Size, appearance and solubility of indomethacin for the nanoemulsions (mean, *n* = 5).
