# Supplementary material for: Development of a New Ex Vivo Lipolysis-Absorption Model for Nanoemulsions
Source: Pharmaceutics. 2019 Apr 4;11(4):164. doi: 10.3390/pharmaceutics11040164 (PMC6523376; doi:10.3390/pharmaceutics11040164)
Supplement: Supplementary file 1 [file pharmaceutics-11-00164-s001.pdf]

pharmaceutics

Supplementary Materials: Development of a New Ex Vivo Lipolysis-Absorption Model for Nanoemulsions

Lu Xiao, Ying Liu and Tao Yi

S1. Screening Formulations of Indomethacin by Pseudo-Ternary Phase Diagrams

The solubilities of indomethacin in various excipients were determined and Labrafac@Lipohile WL1349 (6.91 ± 0.32 mg·g^{-1}), Cremophor RH40 (19.98 ± 0.37 mg·g^{-1}) and Transcutol P (219.76 ± 4.42 mg·g^{-1}) were selected respectively as oil, surfactant and co-surfactant due to their good solubilities. In order to find an optimal range of component proportions to obtain transparent and stable microemulsions, pseudo-ternary phase diagrams were constructed by titration with different surfactant/co-surfactant ratios. The microemulsion areas, which represented the existence field of clear and transparent, stable microemulsions, containing Labrafac@Lipohile WL1349 as oil and the mixing ratio (k_m) of Cremophor RH40 and Transcutol P fixed, respectively, at 3:1, 1:1 and 1:3 (*v*/*v*) in the pseudo-ternary phase-diagrams, were shown in Figure 1. Larger emulsion region was obtained in the system in which k_m was at 3:1, compared to k_m at 1:1 and 1:3. These results indicated that the system containing the mixture of Cremophor RH40/Transcutol P (k_m =3:1) was selected.

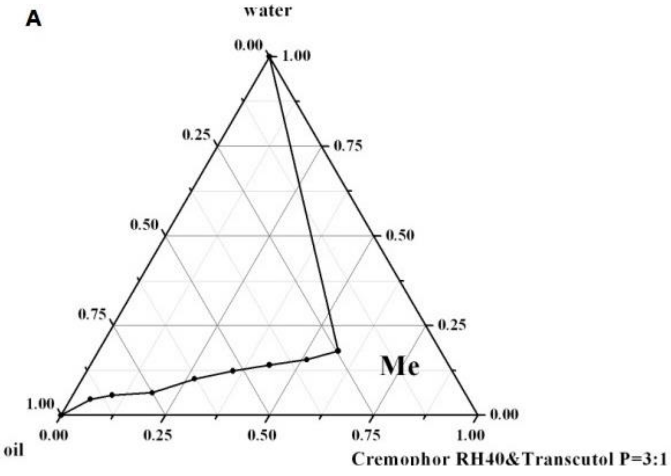

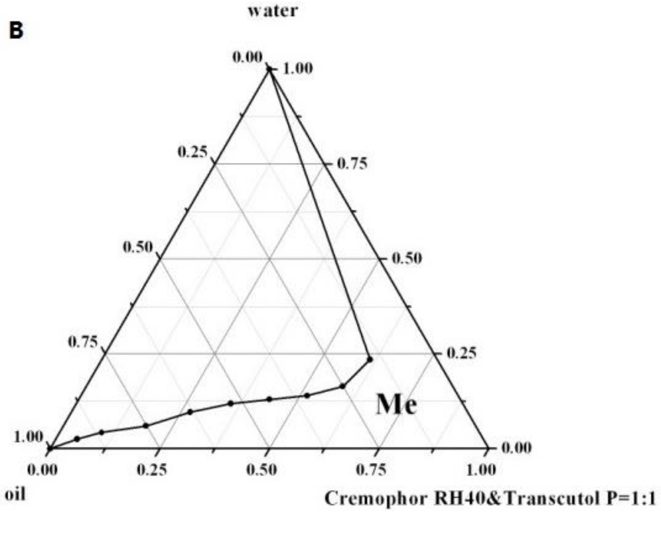

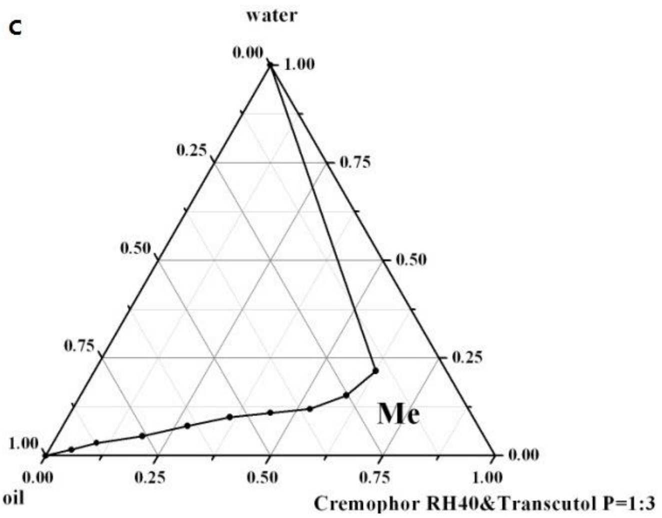

Figure S1. Pseudo-ternary Phase Diagrams: "Me" represented microemulsion area; (**A**) the mixing ratio of Cremophor RH40 and Transcutol P was 3:1; (**B**) the mixing ratio of Cremophor RH40 and Transcutol P was 1:1; (**C**) the mixing ratio of Cremophor RH40 and Transcutol P was 1:3.

S2. Preparation of the Lipid-Based Formulation of Indomethacin

Labrafac@Lipohile WL1349, Cremophor RH40 and Transcutol P (k_m = 3:1) were the basic components for the further studies. The formulation was prepared by dissolving indomethacin in Transcutol P and then mixing with Labrafac@Lipohile WL1349 and Cremophor RH40. Finally, a homogenous mixture was obtained. The particle size, emulsifying efficiency and indomethacin solubility were determined, as shown in Supplementary Table S1. The droplet sizes of four formulations were measured by photon correlation spectroscopy using Zeta-sizer Nano ZS 90 (Malvern Instruments, U.K.). The microscopic droplet of the four nanoemulsions were determined by transmission electron microscopy and showed in Supplementary Figure S2. All samples were diluted with double-distilled water and the average particle size in nanometers was then measured. Emulsifying efficiency was determined by the progressive titration of formulations containing indomethacin at 37 °C with magnetic force stirring. The time of forming stable emulsion was recorded and the appearance of the stable emulsion was observed. According to the results, Formulation II was selected as the optimal one for the smallest droplet size and the better solubility.

Table S1. Self-emulsifying Time, droplet Size, appearance and solubility of indomethacin for the nanoemulsions (mean, *n* = 5).

Formulations	Formulation composition (oil: surfactant: co-surfactant)	Appearance	Droplet Size (nm)	Self-Emulsifying Time (s)	Solubility ($mg \cdot g^{-1}$)
I	10:67.5:22.5	clear	33.11	32.8	59.63
II	20:60:20	clear	29.75	57.3	53.77
III	30:52.5:17.5	slightly bluish	48.51	67.4	47.91
IV	40:45:15	slight less clear	43.00	71.9	42.05

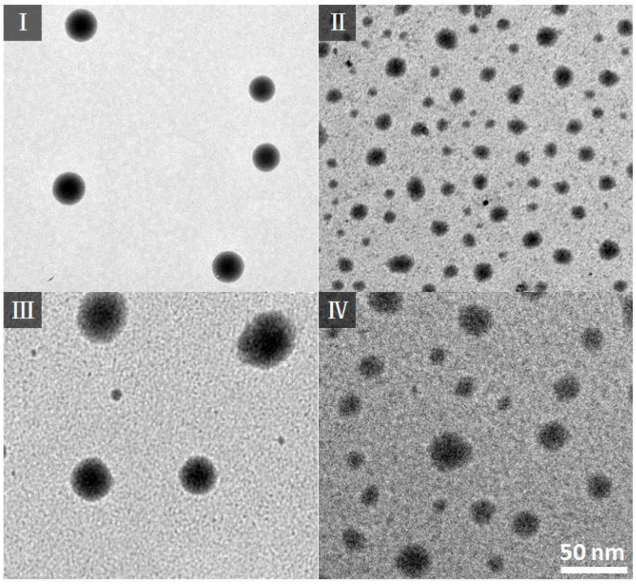

Figure S2. The microscopic images of the four formulations. Formulations were showed as labeled and the ruler was in the bottom right corner of the diagram.
